# Cannabinoid Type 1 and Type 2 Receptor Antagonists Prevent Attenuation of Serotonin-Induced Reflex Apneas by Dronabinol in Sprague-Dawley Rats

**DOI:** 10.1371/journal.pone.0111412

**Published:** 2014-10-28

**Authors:** Michael W. Calik, David W. Carley

**Affiliations:** 1 Center for Narcolepsy, Sleep and Health Research, University of Illinois at Chicago, Chicago, Illinois, United States of America; 2 Department of Biobehavioral Health Science, University of Illinois at Chicago, Chicago, Illinois, United States of America; 3 Department of Medicine, University of Illinois at Chicago, Chicago, Illinois, United States of America; Dalhousie University, Canada

## Abstract

The prevalence of obstructive sleep apnea (OSA) in Americans is 9% and increasing. Increased afferent vagal activation may predispose to OSA by reducing upper airway muscle activation/patency and disrupting respiratory rhythmogenesis. Vagal afferent neurons are inhibited by cannabinoid type 1 (CB_1_) or cannabinoid type 2 (CB_2_) receptors in animal models of vagally-mediated behaviors. Injections of dronabinol, a non-selective CB_1_/CB_2_ receptor agonist, into the nodose ganglia reduced serotonin (5-HT)-induced reflex apneas. It is unknown what role CB_1_ and/or CB_2_ receptors play in reflex apnea. Here, to determine the independent and combined effects of activating CB_1_ and/or CB_2_ receptors on dronabinol’s attenuating effect, rats were pre-treated with CB_1_ (AM251) and/or CB_2_ (AM630) receptor antagonists. Adult male Sprague-Dawley rats were anesthetized, instrumented with bilateral electrodes to monitor genioglossus electromyogram (EMGgg) and a piezoelectric strain gauge to monitor respiratory pattern. Following intraperitoneal treatment with AM251 and/or AM630, or with vehicle, serotonin was intravenously infused into a femoral vein to induce reflex apnea. After baseline recordings, the nodose ganglia were exposed and 5-HT-induced reflex apneas were again recorded to confirm that the nerves remained functionally intact. Dronabinol was injected into each nodose ganglion and 5-HT infusion was repeated. Prior to dronabinol injection, there were no significant differences in 5-HT-induced reflex apneas or phasic and tonic EMGgg before or after surgery in the CB_1_, CB_2_, combined CB_1_/CB_2_ antagonist, and vehicle groups. In the vehicle group, dronabinol injections reduced 5-HT-induced reflex apnea duration. In contrast, dronabinol injections into nodose ganglia of the CB_1_, CB_2_, and combined CB_1_/CB_2_ groups did not attenuate 5-HT-induced reflex apnea duration. However, the CB_1_ and CB_2_ antagonists had no effect on dronabinol’s ability to increase phasic EMGgg. These findings underscore the therapeutic potential of dronabinol in the treatment of OSA and implicate participation of both cannabinoid receptors in dronabinol’s apnea suppression effect.

## Introduction

Sleep-disordered breathing (SDB) is characterized by repeated apnea and hypopnea events [1]. SDB contributes to acute pathophysiological consequences, such as hypoxemia/hypercapnia, fragmented sleep, and exaggerated fluctuations in heart rhythm, blood pressure, and intrathoracic pressure, that can develop into long-term sequelae such as hypertension and other cardiovascular morbidities [1]–[3]. The most prevalent SDB, affecting 14% and 5% of American men and women, respectively, is obstructive sleep apnea (OSA) [1]. Standard treatment for OSA is to pneumatically splint the upper airway using continuous positive airway pressure (CPAP). CPAP is extremely efficacious when used properly; however, CPAP is poorly tolerated [4]. Other mechanical treatments exist, but there are no approved pharmacologic treatments for OSA [5], and efforts to develop such treatments have been hampered by incomplete knowledge of the relevant state-dependent peripheral and central neural mechanisms controlling upper airway muscles.

The vagus nerves are integral peripheral components in respiratory control, carrying important information from the lungs that contributes to reflex responses regulating: tidal volume, respiratory frequency, augmented breaths and bronchoconstriction [6]. The nodose ganglia of the vagus nerves contain receptors for amino acids, monoamines, neuropeptides, and other neurochemicals that, when activated, can modify vagal afferent activity [7]. Decreasing afferent vagal nerve activity by pharmacological intervention increases upper airway activity [8], and ameliorates SDB in rats [9] and bulldogs [10]. Conversely, increasing vagal nerve activity by intraperitoneal (IP) injection of serotonin (5-HT) increases sleep apnea frequency in conscious rats [11]. Similarly, humans with vagus nerve stimulators implanted for refractory epilepsy have increased apnea-hypopnea index during sleep [12].

A recent and novel approach to alleviate OSA is the administration of dronabinol, a nonselective cannabinoid type 1 (CB_1_) and type 2 (CB_2_) receptor agonist. Systemic administration of dronabinol attenuates spontaneous sleep-related apnea in chronically-instrumented conscious rats [13] and in humans with OSA [14]. However, these experiments in chronically-instrumented rats or humans with OSA do not elucidate the mechanisms involved in the amelioration of apnea by dronabinol.

Using a well-established acute rat model of reflex apnea [15], dronabinol injected directly into the nodose ganglia modulated vagal afferents by attenuating 5-HT_3_ receptor-mediated apnea and increasing genioglossus muscle activity [16]. However, it is unknown if attenuation of apnea occurs via CB_1_ or CB_2_ receptors, or both [17]–[21]. The nodose ganglia contain both CB receptors [22], but it is unknown the relative expression levels of these CB receptors on the nodose ganglia. Generally, CB_1_ receptors are more abundant in the nervous system than CB_2_ receptors [23], and CB_1_ receptor knock-out mice display more apneas compared to wild-type controls [24]. Further complicating the role of cannabimimetics in afferent vagal activity is the observation that cannabimimetics can suppress nerve/neuronal activity via mechanisms independent of cannabinoid (CB) receptors. In cultured nodose ganglion cells activated by 5-HT, anandamide attenuated 5-HT-induced currents independent of G protein coupled signaling [25]. Moreover, cannabimimetics like Δ9-tetrahydrocannabinol (Δ9-THC) and anandamide inhibited 5-HT_3_ receptor induced-currents in cultured HEK 293 cells and Xenopus oocytes, cells that lack CB receptors [26], [27]. These studies suggest that CBs can allosterically modulate ionotropic receptors [28].

Here, using the acute rat model of reflex apnea, we hypothesized that the attenuation of 5-HT-induced apnea and the increased upper airway tone produced by nodose ganglion dronabinol injection would be reversed by IP pre-treatment with AM251, a CB_1_ antagonist, but not by pre-treatment AM630, a CB_2_ antagonist.

## Methods

### Ethics statement

All animal studies, procedures, and protocols were approved by the Animal Care Committee of the University of Illinois at Chicago (Protocol no: 11-217).

### Animals

Detailed methods have been previously described [16]. Thirty-six adult male Sprague-Dawley rats (Harlan Laboratories, Indianapolis, IN, USA) were housed in pairs, maintained on a 12∶12 hour light:dark cycle at 22±0.5°C, and given *ad libitum* access to food and water.

### Acute Experimental Paradigm

Rats were anesthetized (initial injection ketamine:xylazine 100∶10 mg/kg). In a balanced design, rats were given IP injections of 15% dimethyl sulfoxide (DMSO) in PBS (1 ml) with AM251 (0.5 mg/kg, n = 6; or 5 mg/kg, n = 6), or AM630 (0.5 mg/kg, n = 6; or 5 mg/kg, n = 6), or AM251/AM630 (5/5 mg/kg, n = 6). Vehicle control rats (n = 6) were given IP injections of 15% dimethyl sulfoxide (DMSO) in PBS (1 ml). The femoral vein was cannulated for 5-HT infusion to induce reflex apneas, and insulated stranded stainless steel wire electrodes were inserted bilaterally into the genioglossus muscles (1 mm lateral to the midline) to monitor genioglossus electromyogram (EMGgg). A piezoelectric strain gauge (Ambu, Glen Burnie, MD, USA) placed around the abdomen was used to monitor respiratory pattern. During recordings, surgical plane of anesthesia was monitored by toe pinch, and if necessary, rats were re-injected with anesthetic (ketamine:xylazine 100∶5 mg/kg).

Before neck surgery, baseline respiratory patterns and EMGgg were recorded from 2–3 reflex apnea responses to 5-HT hydrochloride. Serotonin concentration was 12.5 µg/kg (MP Biomedicals, Solon, OH, USA) in PBS (pH 7.4; 0.35 ml/kg) administered via the cannulated femoral vein using an infusion pump (63 ml/hr; KD Scientific, Holliston, MA, USA). After baseline recordings, nodose ganglia were exposed and cleared of connective tissue. Reflex apneas were recorded to confirm that nerves/ganglia were not damaged during the surgery (surgery baseline recording). After confirmation that nerves/ganglia were functionally intact, rats received dronabinol (100 µg/5 µl sesame oil per ganglion; Mylan Pharmaceuticals, Morgantown, WV, USA) injections directly into the nodose ganglia, and then 5-HT infusions and recordings were repeated (nodose injection recording). Infusions of 5-HT were performed at intervals greater than 5 minutes to prevent tachyphylaxis [15], [16], [29], [30] and to allow for return to baseline of EMG and respiratory pattern.

### Data Processing and Statistical Analysis

During data acquisition, EMGgg and respiratory signals were amplified (CyberAmp, Sunnyvale, CA, USA), band-pass filtered (10–240 Hz and 1–10 Hz, respectively), digitized at 500 Hz and recorded using SciWorks Experimenter software (DataWave Technologies, Loveland, CO, USA), and saved on a personal computer. To process data, EMGgg signals were rectified and smoothed with a time constant of 100 ms using Spike2 software (Cambridge Electronic Design, Cambridge, England). Tonic EMGgg signals were defined as the nadir of smoothed expiratory genioglossus activity. Phasic EMGgg signals were defined as the peak of smoothed inspiratory genioglossus activity minus tonic EMGgg. Breath durations, and phasic and tonic EMGgg amplitudes were averaged from the five previous breaths before 5-HT infusion. Apnea durations were defined as the longest breath duration within 30 seconds following 5-HT infusion. Since each rat received 2–3 infusions of 5-HT under each condition (Baseline, Surgery Baseline, and Nodose Injection), the EMGgg and respiratory pattern from the 2–3 infusions were averaged together for each condition.

For statistical analysis, SigmaStat version 3.11 (Systat Software, Inc., Chicago, IL, USA) was used. Data (mean ± SEM) were analyzed using two-way repeated measures ANOVA (condition×antagonist treatment) with Tukey’s post hoc multiple comparison test. Statistical significance was set at p<0.05.

## Results

### CB_1_ and CB_2_ Antagonists Reverse Dronabinol’s Attenuation of 5-HT-induced Apnea

**Figure 1:**
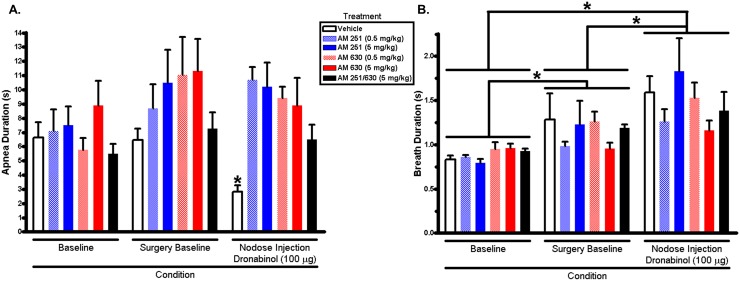
Apnea and breath durations quantified from acute 5-HT-induced apnea experiments. (**A**) Dronabinol (100 µg) injected into the nodose ganglia significantly attenuated apnea duration. Intraperitoneal injection of cannabinoid type 1 (AM2521), or cannabinoid type 2 (AM630), receptor antagonists, or both, reversed dronabinol’s apnea attenuation. *p<0.05 compared to surgery baseline recording, Tukey’s post hoc multiple comparison test. (**B**) Dronabinol injected into the nodose ganglia did not have any effect on breath duration. However, there was a significant (p<0.05, two-way repeated measures ANOVA) main effect of condition on breath duration; *p<0.05, Tukey’s post hoc multiple comparison test.

Figure 1 demonstrates apnea duration (Fig. 1A) and breath duration (Fig. 1B) of respiratory pattern recordings from rats pre-treated with AM251, AM630, combined AM251/AM630, and vehicle. There was a significant interaction between the main effects of antagonist treatment and condition on length of 5-HT-induced apnea duration (Fig. 1A; F_10, 60_ = 2.35, p = 0.02). Post hoc analysis revealed that rats with no IP pre-treatment with CB antagonist (vehicle control) had significantly attenuated apneas after local injection of dronabinol (nodose injection recording) compared to baseline (p = 0.03) and surgery baseline recordings (p = 0.04). These data replicate past findings of locally administered dronabinol’s capacity to attenuate reflex apnea [16]. CB_1_ or CB_2_ receptor antagonist at both concentrations (0.5 or 5 mg/kg), or combined CB_1_/CB_2_ antagonist, pre-treatment reversed dronabinol’s attenuation of reflex apnea.

Similar to past observations [16], there was only a main effect of condition on breath duration (Fig. 1B; F_2, 60_ = 2.35, p<0.01), characterized by a progressive increase in breath duration through the recordings (baseline < surgery baseline < nodose injection). Post hoc analysis revealed increases in breath duration from baseline to surgery baseline (p = 0.02), and from surgery baseline to nodose injections (p<0.01). The increased breath duration was the result of surgery or from the effects of anesthesia.

CB_1_ and CB_2_ Antagonists Did Not Reverse Dronabinol’s Increases in Phasic EMGgg.

**Figure 2:**
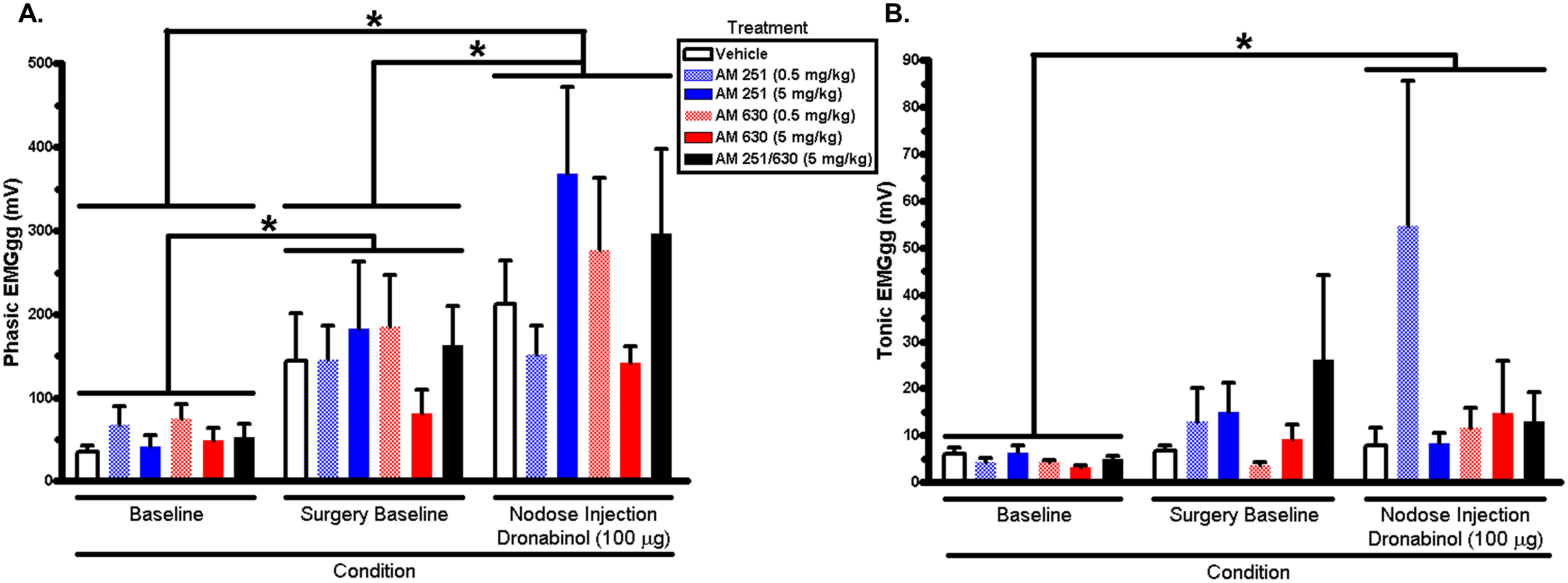
Phasic (A) and tonic (B) genioglossus electromyogram amplitude (mV) quantified from acute 5-HT-induced apnea experiments. There was a significant (p<0.05, two-way repeated measures ANOVA) main effect of condition on phasic and tonic genioglossus activity; *p<0.05, Tukey’s post hoc multiple comparison test. EMGgg = genioglossus electromyogram.

Figure 2 illustrates data of phasic (Fig. 2A) and tonic (Fig. 2B) genioglossus muscle activity from EMGgg recordings from IP pretreated rats with antagonists or vehicle that had dronabinol locally injected into the nodose ganglia. Consistent with past observations [16], dronabinol injected in the nodose ganglia increased phasic EMGgg across the conditions (Fig. 2A; condition main effect: F_2, 60_ = 33.4, p<0.01). Post hoc tests revealed that pre-treatment with CB antagonists failed to attenuate the increase in phasic EMGgg (Baseline > Surgery Baseline > Nodose injection, p<0.01). Interestingly, there also was a main effect of condition on tonic EMG (Fig. 2B; condition main effect: F_2, 60_ = 3.53, p = 0.04) Post hoc tests revealed that nodose injection tonic EMGgg signals were significantly increased compared to baseline EMGgg signals (p = 0.03).

## Discussion

Poor patient adherence to CPAP for the treatment of OSA underscores the need to develop better treatment options [4]. Combined with increasing prevalence of OSA and its associated comorbidities [1]–[3], tolerable and easily applicable treatments for OSA, like pharmacotherapies, must be developed. However, decades of unsuccessful efforts to identify effective drug treatments for OSA [5] highlight the need for novel and innovative approaches.

Dronabinol, a non-selective CB agonist, has been shown to decrease sleep apnea in conscious rats [13] and in humans with OSA [14]. In an acute rat model of reflex apnea, 5-HT-induced apnea was attenuated or blocked and phasic EMG increased in rats receiving nodose ganglia injections of dronabinol [16], suggesting that modulating vagal afferent activity has an important role in respiratory control [6]. Here, we show that dronabinol modulates vagal afferents through both CB_1_ and CB_2_ receptors. By blocking these receptors through IP pre-treatment with CB antagonists, we reversed the attenuation of reflex apnea produced by nodose ganglia injections of dronabinol.

The concept of pharmacologically modulating vagal afferent activity to treat sleep apnea has been employed previously. Yoshioka *et al.* first showed that modulation of vagal afferents by antagonism of 5-HT_3_ receptors in acute experiments in rats attenuated reflex apnea [15]. This observation was then extended to spontaneous sleep-related apnea by Radulovacki *et al.* who showed in a chronically-instrumented conscious rats that 5-HT_3_ antagonism decreased sleep apnea frequency [9]. Fenik and colleagues suggested that peripheral antagonism of 5-HT_3_ receptors on vagus nerves augments inspiratory drive to hypoglossal nerves in anesthetized, paralyzed, and mechanically ventilated rats [8]. These three studies together provided a rationale for testing 5-HT antagonists in a model of obstructive sleep apnea. Indeed, Veasey *et al.* demonstrated that 5-HT_3_ antagonism reduced the respiratory disturbance index during sleep in the English bulldog [10]. In humans, a serotonin antagonist combined with a selective serotonin reuptake inhibitor decreased apnea-hypopnea index during sleep, but the therapeutic window for this effect appears to be narrow [5], [31]. Thus, modulating vagal afferent activity to treat sleep apnea has potential if the correct pharmacotherapy can be applied.

CBs have been used to modulate vagal afferent activity via the inhibitory G protein-coupled (G_i/o_) CB_1_ and CB_2_ receptors located on the nodose ganglia [22]. Vagal afferent modulation via CB receptors has been suggested as a treatment not just for sleep apnea [13], [14], but also for emesis [17]–[19] and for chronic cough [20], [21]. In the vagally-mediated behavior of emesis, CB_1_ receptors are implicated. In contrast, in the vagally-mediated behavior of chronic cough, CB_2_ receptors are involved. We hypothesized that dronabinol’s attenuation of reflex apnea occurred via CB_1_ receptors, and not CB_2_ receptors, because CB_1_ receptors are more abundant in the nervous system than CB_2_ receptors [23]. Moreover, CB_1_ receptor knock-out mice display more sleep apneas than wild-type controls, suggesting CB tone is important in respiratory stability [24]. However, in our study, when either CB receptor subtype was antagonized, or when both were antagonized, by IP pre-treatment, reflex apnea attenuation by local injection of dronabinol was reversed. CB_1_ and CB_2_ receptors are positively coupled to A-type potassium channels [32], which are expressed on nodose ganglion cells and are known to decrease nodose ganglion/vagus nerve excitability [33]–[35]. It is possible that activating only one of the two CB receptor subtypes is insufficient to initiate a G_i/o_ cascade that overcomes 5-HT-induced excitability of the vagus nerve. In contrast, activation of either CB_1_ or CB_2_ receptor centrally had an antiemetic effect in brainstem-induced emesis [36]. The difference in effects of CB antagonism on vagal afferents seen in reflex apnea compared to vagally-mediated emesis [17]–[19] or chronic cough [20], [21] could be due to differential receptor expression on A- and/or C-fibers of the vagus nerves/nodose ganglia. In other words, there might be differences in CB receptor expression profiles between the vagally-mediated behaviors of apnea, emesis, and cough. Less than 60% of nodose ganglia cells exhibit CB_1_ expression [37], and though CB_2_ receptors are located on vagal afferents [22], no studies have been conducted to quantify to CB_2_ receptors expression; however, there is differential expression of other receptor types on cell bodies in the nodose ganglia [7]. It is possible that the vagal afferents responsible for emesis and chronic cough express only one of the two subtypes of CB receptors, while vagal afferents responsible for sleep apnea express both CB receptor subtypes. Studies to look at CB receptor expression in the nodose ganglia would be of interest.

CBs have been reported to allosterically modulate many different types of ionotropic receptors [28], including 5-HT receptors on nodose ganglion cells. Anandamide, a cannabimimetic, attenuated 5-HT-induced currents in cultured nodose ganglion cells independent of G protein coupled signaling [25]. In cultured HEK 293 cells and Xenopus oocytes, cells that lack CB receptors, Δ9-THC and anandamide inhibited 5-HT-induced currents [26], [27]. Barann and colleagues also showed that 9-THC reduced 5-HT-induced current in dissociated rat nodose ganglion cells [26]. However, no CB receptor antagonists were used in these electrophysiological experiments, so determination of whether the current reduction was attributable to CB receptor dependent or independent mechanisms is unknown. However, in the present work, CB antagonism was sufficient to reverse dronabinol’s effect on reflex apnea, arguing against dronabinol acting via CB receptor-independent mechanisms. However, Yang and colleagues showed that allosteric inhibition of the 5-HT_3_ receptor via cannabidiol, a cannabimimetic, is dependent on expression levels 5-HT_3_ receptor [27]. It could be that the high expression levels of 5-HT_3_ on nodose ganglion cells [7] and the dose of dronabinol used to inject into the nodose ganglia is insufficient to allosterically inhibit 5-HT_3_ receptors, thus leaving the activation of CB receptors as the main inhibitory signal of vagal afferents. Patch clamping electrophysiology of nodose ganglion cells using CB receptor antagonisms need to be conducted to resolve this issue.

In contrast to CB antagonism reversing dronabinol’s attenuation of reflex apnea, CB antagonism did not reverse dronabinol-induced increases in phasic or tonic genioglossus EMG. Three explanations can account for this observation. The first possibility is that brainstem respiratory control of the hypoglossal motoneurons responsible for genioglossus activity is distinct from that of the phrenic motoneurons responsible for controlling the diaphragm [38]–[40]. Vagal afferents from pulmonary and upper airway tissues synapse in the nucleus of the solitary tract (NTS) [41], [42], which is part of the respiratory circuitry including the ponto-medullary pattern generator that projects to the phrenic and hypoglossal motor nuclei [43]. Different areas of the NTS stimulated with L-glutamate lead to different patterns of respiratory responses [42], and CB receptors are located presynaptically on first-order glutamatergic vagal afferent neurons and on second-order GABAergic neurons in NTS [44], [45]. It is feasible that systemic pre-treatment with CB receptor antagonists can increase genioglossus activity through modulation of first- and/or second-order NTS neurons that project to hypoglossal motoneurons, but does not affect phrenic motoneurons. A second possibility is that antagonism of CB receptors on hypoglossal motoneurons leads to postsynaptic potentiation [46]. It is possible that systemic pre-treatment with CB receptor antagonists can increase genioglossus activity by directly increasing hypoglossal nerve activity. It remains to be seen how central antagonism of CB receptors differentially modulates genioglossus and diaphragmatic activity.

A final possible explanation of the observation that CB antagonism did not reverse dronabinol-induced increases in EMGgg is that vagal afferent neurons that project to circuitry responsible for upper airway activity might not contain CB receptors [37], and therefore are not affected by CB receptor antagonism. These vagal afferents are then only inhibited or attenuated by allosteric modulation of the 5-HT_3_ receptor [26], [27]. Fenik and colleagues have shown that antagonizing 5-HT_3_ receptors peripherally with ondansetron increased inspiratory modulation of hypoglossal nerve activity [8]. This could explain the increases in phasic/tonic EMGgg seen in this study.

In summary, we show that CB_1_ and CB_2_ receptor antagonists reversed the suppression of 5-HT-induced apnea by locally-injected dronabinol into the nodose ganglia, but did not reverse increases phasic activation of the genioglossus. These data support the view that systemic dronabinol stabilizes respiratory pattern, in part, by acting via CB_1_ and CB_2_ receptors at the nodose ganglia. These findings underscore the therapeutic potential of dronabinol in the treatment of OSA and implicate participation of both cannabinoid receptors in dronabinol’s apnea suppression effect.

## References

[1] Peppard PE, Young T, Barnet JH, Palta M, Hagen EW, et al (2013) Increased Prevalence of Sleep-Disordered Breathing in Adults. Am J Epidemiol 177: 1006–1014. 23589584 10.1093/aje/kws342 PMC3639722

[2] Somers VK, White DP, Amin R, Abraham WT, Costa F, et al (2008) Sleep apnea and cardiovascular disease: an American Heart Association/american College Of Cardiology Foundation Scientific Statement from the American Heart Association Council for High Blood Pressure Research Professional Education Committee, Council on Clinical Cardiology, Stroke Council, and Council On Cardiovascular Nursing. In collaboration with the National Heart, Lung, and Blood Institute National Center on Sleep Disorders Research (National Institutes of Health). Circulation 118: 1080–1111. 18725495 10.1161/CIRCULATIONAHA.107.189375

[3] Shamsuzzaman AS, Gersh BJ, Somers VK (2003) Obstructive sleep apnea: implications for cardiac and vascular disease. Jama 290: 1906–1914. 14532320 10.1001/jama.290.14.1906

[4] Weaver TE, Grunstein RR (2008) Adherence to continuous positive airway pressure therapy: the challenge to effective treatment. Proc Am Thorac Soc 5: 173–178. 18250209 10.1513/pats.200708-119MG PMC2645251

[5] Mason M, Welsh EJ, Smith I (2013) Drug therapy for obstructive sleep apnoea in adults. Cochrane Database Syst Rev 5: CD003002. 10.1002/14651858.CD003002.pub3 PMC11623339 23728641

[6] Kaczynska K, Szereda-Przestaszewska M (2013) Nodose ganglia-modulatory effects on respiration. Physiol Res 62: 227–235. 23489183 10.33549/physiolres.932412

[7] Zhuo H, Ichikawa H, Helke CJ (1997) Neurochemistry of the nodose ganglion. Prog Neurobiol 52: 79–107. 9185234 10.1016/s0301-0082(97)00003-8

[8] Fenik P, Ogawa H, Veasey SC (2001) Hypoglossal nerve response to 5-HT3 drugs injected into the XII nucleus and vena cava in the rat. Sleep 24: 871–878. 11766156 10.1093/sleep/24.8.871

[9] Radulovacki M, Trbovic SM, Carley DW (1998) Serotonin 5-HT3-receptor antagonist GR 38032F suppresses sleep apneas in rats. Sleep 21: 131–136. 9542796 10.1093/sleep/21.2.131

[10] Veasey SC, Chachkes J, Fenik P, Hendricks JC (2001) The effects of ondansetron on sleep-disordered breathing in the English bulldog. Sleep 24: 155–160. 11247051 10.1093/sleep/24.2.155

[11] Carley DW, Radulovacki M (1999) Role of peripheral serotonin in the regulation of central sleep apneas in rats. Chest 115: 1397–1401. 10334159 10.1378/chest.115.5.1397

[12] Parhizgar F, Nugent K, Raj R (2011) Obstructive sleep apnea and respiratory complications associated with vagus nerve stimulators. J Clin Sleep Med 7: 401–407. 21897779 10.5664/JCSM.1204 PMC3161774

[13] Carley DW, Paviovic S, Janelidze M, Radulovacki M (2002) Functional role for cannabinoids in respiratory stability during sleep. Sleep 25: 391–398. 12071539

[14] Prasad B, Radulovacki MG, Carley DW (2013) Proof of concept trial of dronabinol in obstructive sleep apnea. Front Psychiatry 4: 1. 23346060 10.3389/fpsyt.2013.00001 PMC3550518

[15] Yoshioka M, Goda Y, Togashi H, Matsumoto M, Saito H (1992) Pharmacological characterization of 5-hydroxytryptamine-induced apnea in the rat. J Pharmacol Exp Ther 260: 917–924. 1531363

[16] Calik MW, Radulovacki M, Carley DW (2014) Intranodose ganglion injections of dronabinol attenuate serotonin-induced apnea in Sprague-Dawley rat. Respir Physiol Neurobiol 190: 20–24. 24121138 10.1016/j.resp.2013.10.001 PMC3880550

[17] Darmani NA, Johnson JC (2004) Central and peripheral mechanisms contribute to the antiemetic actions of delta-9-tetrahydrocannabinol against 5-hydroxytryptophan-induced emesis. Eur J Pharmacol 488: 201–212. 15044052 10.1016/j.ejphar.2004.02.018

[18] Darmani NA (2001) Delta-9-tetrahydrocannabinol differentially suppresses cisplatin-induced emesis and indices of motor function via cannabinoid CB(1) receptors in the least shrew. Pharmacology, biochemistry, and behavior 69: 239–249. 10.1016/s0091-3057(01)00531-7 11420092

[19] Ray AP, Griggs L, Darmani NA (2009) Delta 9-tetrahydrocannabinol suppresses vomiting behavior and Fos expression in both acute and delayed phases of cisplatin-induced emesis in the least shrew. Behav Brain Res 196: 30–36. 18721829 10.1016/j.bbr.2008.07.028 PMC2613838

[20] Belvisi MG, Patel HJ, Freund-Michel V, Hele DJ, Crispino N, et al (2008) Inhibitory activity of the novel CB2 receptor agonist, GW833972A, on guinea-pig and human sensory nerve function in the airways. Br J Pharmacol 155: 547–557. 18695648 10.1038/bjp.2008.298 PMC2579660

[21] Patel HJ, Birrell MA, Crispino N, Hele DJ, Venkatesan P, et al (2003) Inhibition of guinea-pig and human sensory nerve activity and the cough reflex in guinea-pigs by cannabinoid (CB2) receptor activation. Br J Pharmacol 140: 261–268. 12970104 10.1038/sj.bjp.0705435 PMC1574031

[22] Rohof WO, Aronica E, Beaumont H, Troost D, Boeckxstaens GE (2012) Localization of mGluR5, GABAB, GABAA, and cannabinoid receptors on the vago-vagal reflex pathway responsible for transient lower esophageal sphincter relaxation in humans: an immunohistochemical study. Neurogastroenterol Motil 24: 383–e173. 22256945 10.1111/j.1365-2982.2011.01868.x

[23] Onaivi ES, Ishiguro H, Gu S, Liu QR (2012) CNS effects of CB2 cannabinoid receptors: beyond neuro-immuno-cannabinoid activity. Journal of psychopharmacology (Oxford, England) 26: 92–103. 10.1177/0269881111400652 PMC3388033 21447538

[24] Silvani A, Berteotti C, Bastianini S, Cohen G, Lo Martire V, et al (2014) Cardiorespiratory anomalies in mice lacking CB1 cannabinoid receptors. PloS one 9: e100536. 24950219 10.1371/journal.pone.0100536 PMC4065065

[25] Fan P (1995) Cannabinoid agonists inhibit the activation of 5-HT3 receptors in rat nodose ganglion neurons. J Neurophysiol 73: 907–910. 7760148 10.1152/jn.1995.73.2.907

[26] Barann M, Molderings G, Bruss M, Bonisch H, Urban BW, et al (2002) Direct inhibition by cannabinoids of human 5-HT3A receptors: probable involvement of an allosteric modulatory site. Br J Pharmacol 137: 589–596. 12381672 10.1038/sj.bjp.0704829 PMC1573528

[27] Yang KH, Isaev D, Morales M, Petroianu G, Galadari S, et al (2010) The effect of Delta9-tetrahydrocannabinol on 5-HT3 receptors depends on the current density. Neuroscience 171: 40–49. 20800662 10.1016/j.neuroscience.2010.08.044

[28] Pertwee RG (2008) The diverse CB1 and CB2 receptor pharmacology of three plant cannabinoids: delta9-tetrahydrocannabinol, cannabidiol and delta9-tetrahydrocannabivarin. Br J Pharmacol 153: 199–215. 17828291 10.1038/sj.bjp.0707442 PMC2219532

[29] Ginzel KH, Kottegoda SR (1954) The action of 5-hydroxytryptamine and tryptamine on aortic and carotid sinus receptors in the cat. The Journal of physiology 123: 277–288. 13143510 10.1113/jphysiol.1954.sp005050 PMC1366201

[30] Nishi K (1975) The action of 5-hydroxytryptamine on chemoreceptor discharges of the cat’s carotid body. Br J Pharmacol 55: 27–40. 1182345 10.1111/j.1476-5381.1975.tb07606.x PMC1666726

[31] Prasad B, Radulovacki M, Olopade C, Herdegen JJ, Logan T, et al (2010) Prospective trial of efficacy and safety of ondansetron and fluoxetine in patients with obstructive sleep apnea syndrome. Sleep 33: 982–989. 20614859 10.1093/sleep/33.7.982 PMC2894441

[32] Svizenska I, Dubovy P, Sulcova A (2008) Cannabinoid receptors 1 and 2 (CB1 and CB2), their distribution, ligands and functional involvement in nervous system structures–a short review. Pharmacology, biochemistry, and behavior 90: 501–511. 10.1016/j.pbb.2008.05.010 18584858

[33] Doan TN, Kunze DL (1999) Contribution of the hyperpolarization-activated current to the resting membrane potential of rat nodose sensory neurons. The Journal of physiology 514 (Pt 1): 125–138. 10.1111/j.1469-7793.1999.125af.x PMC2269051 9831721

[34] Doan TN, Stephans K, Ramirez AN, Glazebrook PA, Andresen MC, et al (2004) Differential distribution and function of hyperpolarization-activated channels in sensory neurons and mechanosensitive fibers. J Neurosci 24: 3335–3343. 15056713 10.1523/JNEUROSCI.5156-03.2004 PMC6730026

[35] Glazebrook PA, Ramirez AN, Schild JH, Shieh CC, Doan T, et al (2002) Potassium channels Kv1.1, Kv1.2 and Kv1.6 influence excitability of rat visceral sensory neurons. The Journal of physiology 541: 467–482. 12042352 10.1113/jphysiol.2001.018333 PMC2290329

[36] Van Sickle MD, Duncan M, Kingsley PJ, Mouihate A, Urbani P, et al (2005) Identification and functional characterization of brainstem cannabinoid CB2 receptors. Science 310: 329–332. 16224028 10.1126/science.1115740

[37] Burdyga G, Varro A, Dimaline R, Thompson DG, Dockray GJ (2010) Expression of cannabinoid CB1 receptors by vagal afferent neurons: kinetics and role in influencing neurochemical phenotype. American journal of physiology 299: G63–69. 20430875 10.1152/ajpgi.00059.2010 PMC2904113

[38] Peever JH, Mateika JH, Duffin J (2001) Respiratory control of hypoglossal motoneurones in the rat. Pflugers Arch 442: 78–86. 11374072 10.1007/s004240000502

[39] Saito Y, Ezure K, Tanaka I (2002) Difference between hypoglossal and phrenic activities during lung inflation and swallowing in the rat. The Journal of physiology 544: 183–193. 12356891 10.1113/jphysiol.2002.022566 PMC2290563

[40] Tadjalli A, Duffin J, Peever J (2010) Identification of a novel form of noradrenergic-dependent respiratory motor plasticity triggered by vagal feedback. J Neurosci 30: 16886–16895. 21159960 10.1523/JNEUROSCI.3394-10.2010 PMC6634916

[41] Hermes SM, Colbert JF, Aicher SA (2014) Differential content of vesicular glutamate transporters in subsets of vagal afferents projecting to the nucleus tractus solitarii in the rat. The Journal of comparative neurology 522: 642–653. 23897509 10.1002/cne.23438 PMC3877231

[42] Marchenko V, Sapru HN (2000) Different patterns of respiratory and cardiovascular responses elicited by chemical stimulation of dorsal medulla in the rat. Brain Res 857: 99–109. 10700557 10.1016/s0006-8993(99)02377-x

[43] Haji A, Takeda R, Okazaki M (2000) Neuropharmacology of control of respiratory rhythm and pattern in mature mammals. Pharmacol Ther 86: 277–304. 10882812 10.1016/s0163-7258(00)00059-0

[44] Chen SW, Wu BY, Xu SP, Fan KX, Yan L, et al (2012) Suppression of CB1 cannabinoid receptor by lentivirus mediated small interfering RNA ameliorates hepatic fibrosis in rats. PloS one 7: e50850. 23251393 10.1371/journal.pone.0050850 PMC3520929

[45] Khlaifia A, Farah H, Gackiere F, Tell F (2013) Anandamide, cannabinoid type 1 receptor, and NMDA receptor activation mediate non-Hebbian presynaptically expressed long-term depression at the first central synapse for visceral afferent fibers. J Neurosci 33: 12627–12637. 23904599 10.1523/JNEUROSCI.1028-13.2013 PMC6618553

[46] Mukhtarov M, Ragozzino D, Bregestovski P (2005) Dual Ca2+ modulation of glycinergic synaptic currents in rodent hypoglossal motoneurones. The Journal of physiology 569: 817–831. 16123105 10.1113/jphysiol.2005.094862 PMC1464266

